# Microarray Based Functional Analysis of Myricetin and Proteomic Study on Its Anti-Inflammatory Property

**DOI:** 10.1155/2019/3746326

**Published:** 2019-03-07

**Authors:** Tao Li, Jihe Zhu, Fangming Deng, Weiguo Wu, Zhibing Zheng, Chenghao Lv, Yong Li, Wei Xiang, Xiangyang Lu, Si Qin

**Affiliations:** ^1^Core Research Program 1515, Key Laboratory for Food Science and Biotechnology of Hunan Province, College of Food Science and Technology, Hunan Agricultural University, Changsha 410128, China; ^2^Hunan Co-Innovation Center for Utilization of Botanical Functional Ingredients, College of Bioscience and Biotechnology, Hunan Agricultural University, Changsha 410128, China; ^3^The United Graduate School of Agricultural Sciences, Faculty of Agriculture, Kagoshima University, Korimoto 1-21-24, Kagoshima 890-0065, Japan

## Abstract

Myricetin has been reported as a promising chemopreventive compound with multiple biofunctions. To evaluate its influence on gene expressions in genome-wide set and further investigate its anti-inflammatory property, the present study performed Gene Ontology and Ingenuity Pathway Analysis (IPA) to describe the basic gene expression characteristics by myricetin treatment in HepG2 cells, confirmed its multi-biofunction by real-time fluorescent quantitative PCR (RT-qPCR), and further verified its anti-inflammatory property by Western blotting and bio-plex-based cytokines assay. The IPA data showed that 337 gene expressions (48% of the top molecules) are disturbed over 2-fold, and the most possible biofunctions of myricetin are the effect on “cardiovascular disease, metabolic disease, and lipid metabolism,” via regulation of 28 molecules with statistic score of 46. RT-qPCR data confirmed the accuracy of microarray data, and cytokines assay results indicated that 6 of the total 27 inflammatory cytokine secretions were significantly inhibited by myricetin pretreatment, including TNF-*α*, IFN-*γ*, IL-1*α*, IL-1*β*, IL-2, and IL-6. The present study is the first time to elucidate the multi-function of myricetin in genome-wide set by IPA analysis and verify its anti-inflammatory property by proteomics of cytokines assay. Therefore, these results enrich the comprehensive bioactivities of myricetin and reveal that myricetin has powerful anti-inflammatory property, which provides encouragement for* in vivo *studies to verify its possible health benefits.

## 1. Introduction

Myricetin is a well-defined natural flavonoid with hydroxyl groups at the positions of 3, 5, 7, 3', 4', and 5', which is widely existed in vegetables, fruits, and teas, as well as medicinal herbs [1]. Myricetin has been proved to be the most potent and promising chemopreventive compound for its multiple biofunctions, such as antioxidation, anti-inflammation, antitumor, anti-diabetes, and anti-mutation effects [2, 3]. For the antioxidant property, myricetin was reported to attenuate the deleterious effect of oxidative stress in human red blood cells [4], prevent against I/R-induced myocardial injury in rats [5], and exhibit a significant hepatoprotective activity to reduce hepatic oxidative stress in mice [6], by the induction of antioxidant genes or proteins. For the anti-inflammatory property, overexpression of tumor necrosis factor-alpha (TNF-*α*) and cyclooxygenase-2 (COX-2) in mouse liver was found to be reduced by myricetin treatment [6], and the production of proinflammatory mediators was inhibited by myricetin via inhibition of NF-*κ*B and STAT1 pathways and induction of Nrf2-HO-1 pathway in LPS-stimulated RAW264.7 macrophages [7]. For the anticancer property, myricetin was recognized to be able to induce apoptosis of Human T24 bladder cancer cells via modulation of Bcl-2 family proteins and caspase-3, significantly inhibit the tumor growth on T24 bladder cancer xenografts model [8], and promote apoptosis through regulation of apoptotic protein Bax, Bad, and Bcl-2 in HepG2 cells [9].

The recent arising concept of nutrigenomics is defined to investigate the omics-wide influences of classical nutrients or other dietary bioactive components in food. Nutrients or dietary bioactive components are mediators that can be detected by the cellular sensor systems and influence gene expressions, protein synthesis, and metabolite production [10]. Nutrigenomics is designed to investigate the dietary components in distinguished cells, tissues, and organisms and to elucidate how they influence the redox balance and homeostasis of the cells, which plays crucial role on human health maintenance and disease prevention [10, 11]. However, limited researches were performed to study the functional effects of flavonoids and elucidate the underlying mechanism by using methods of nutrigenomics. For instance, by using quantitative proteomics approach, EGCG was reported to exert its effect on alleviation to vanadium stress in laying hens through regulation of metal-binding mediation, cell proliferation, and immune function-related proteins [12]. Grape polyphenols were found to improve cellular parameters and reduce the amount of lipogenesis and glycolysis enzymes, enhance fatty acid oxidation and stimulate insulin signaling, and ameliorate protein oxidation or endoplasmic reticulum stress [13]. By using transcriptomics expression analysis, cocoa polyphenols were reported to possess antiobesity effects in high fat diet induced obese rats by regulation of lipid metabolism genes and reduce adiposity in adipose tissue [14]. Recently, a comprehensive review had summarized the data in the last decade and found that dietary polyphenols may function as ideal modulators of the mammalian gene expressions by histone deacetylation, histone acetylation, and DNA methylation in experimental models [15, 16]. Another interesting review further provides the reliable scientific data to reveal the importance of polyphenols to fight against carcinogenesis epigenetically and focuses on the effects of dietary polyphenols to mitigate carcinogenesis [17]. Our previous study had applied microarray to analyze the effects of myricetin on genome-wide set; however, the analysis of the data remains scarce and shallow [18]. Therefore, further analysis on the microarray data of myricetin and other attached new omics tools should be performed, such as proteomics assay.

The studies on the biofunction of polyphenols mainly focus on the regulation of redox signaling pathways and related genes or proteins linked to human health, especially on the inhibition of inflammation [19]. Inflammation is defined by the increased acute phase reactants or other mediators, the activation of inflammatory signal pathways, and abnormal cytokine or inflammatory marker expressions [20]. Cytokines are important inflammatory signaling proteins to mediate a wide range of physiological responses and a spectrum of related diseases, such as tumor growth, infections, and Parkinson's disease. Cytokines are generally detected by either immunoassay or bioassay [21, 22]. Recently, the newly discovered Bio-Plex suspension array instrument from Bio-Rad Company can simultaneously detect up to 100 cytokines in a single well of a 96-well microplate by application of novel technology with color-coded beads. Therefore, bio-plex based technology is of great significance to understand the whole inflammatory process. To elucidate the more detail about the multiple biofunctions of myricetin, comprehensive analysis of microarray data in genome-wide set by advanced software and specific verification of the anti-inflammatory property by proteomic tool were performed in the present study.

## 2. Materials and Methods

### 2.1. Materials and Antibodies

Myricetin (≥99%, purified by HPLC) was purchased from Extrasynthese (Lyon Nord, France). Human hepatoblastoma (HepG2) cells were donated by the Cancer Cell Repository (Tohoku University, Japan). Fetal bovine serum (FBS) and Dulbecco's modified Eagle's medium (DMEM) were purchased from Hyclone (Logan, USA). Lipopolysaccharide (LPS) and other general reagents used in the chemical analysis were purchased from Sigma-Aldrich (Shanghai, China). The antibodies for COX-2, iNOS, *α*-tubulin, and others were purchased from Santa Cruz Biotechnology (Santa Cruz, USA) and Cell Signaling Technology (Beverly, USA).

### 2.2. Reverse Transcription and Real-Time qPCR

HepG2 cells were planted in dishes for 24 h and then treated with 20 *μ*M of myricetin in 0.1% DMSO or alone for additional 9 h. Total RNA was extracted with RNA extraction kit (Nippon Gene Co., Japan) as described accordingly. The primers used in the present study were designed by PRIMER3 and synthesized by company as follows:* AKR1C1*, forward (5'- ATC CCT CCG AGA AGA ACC AT-3') and reverse (5'- ACA CCT GCA CGT TCT GTC TG-3');* AKR1C2*, forward (5'- GAT CCC ATC GAG AAG AAC CA-3') and reverse (5'-ACA CCT GCA CGT TCT GTC TG-3');* GCLC*, forward (5'- GAG CTG GGA GGA AAC CAA G -3') and reverse (5'- TGG TTT GGG TTT GTC CTT TC-3');* SERPINE*, forward (5'-GTG CTG GTG AAT GCC CTC T-3') and reverse (5'- GCA GTT CCA GGA TGT CGT -3');* IL11*, forward (5'-GCG GAC AGG GAA GGG TTA AAG-3') and reverse (5'- GGG CGA CAG CTG TAT CTG G -3');* IGFBP1*, forward (5'- ATG ATG GCT CGA AGG CTC TC-3') and reverse (5'-ATG TCT CAC ACT GTC TGC TGT-3'). Reverse transcription and real-time qPCR were performed with RT-qPCR Kit (Finnzymes Oy., Espoo, Finland) accordingly. Briefly, 200 ng of RNA was reverse-transcribed to cDNA at 37°C for 30 min, and the reaction was then terminated at 85°C for 5 min, and other reaction conditions or procedures were described previously [18]. The results were presented as the relative expression levels normalized with that in control cells.

### 2.3. Microarray Data Analysis and Network Generation

Microarray results were classified by Gene Ontology ID (http://www.geneontology.org/) and analyzed by general method and then were imported into Ingenuity Pathway Analysis (IPA) System (http://www.ingenuity.com) for further analysis. The gene ontology classification and analysis are primary bioinformatics to standardize their presentation of gene and gene product attributes from different species and databases. The Ingenuity Knowledge Base is built by the huge data extracted from the millions of full text literatures with weekly update, which is the leader of its kind to analyze the results originated from microarray. [23]. The original microarray data was imported into the IPA system according to gene accession numbers and the fold change upon myricetin treatment versus control and other necessary data. P<0.002 was set as the cutoff point of the IPA system, and the genes were classified according to the molecular functions. The canonical pathways analysis was performed to identify the most possible disturbed pathways from the IPA system with the most significance in the dataset [24, 25].

The network analysis was carried out to identify series of genes belonging to certain genetic networks associated with functions or diseases in the IPA system and were ranked by the score that represents the possibility that a class of genes were disturbed. The genetic network analysis was also based on the information or findings originated from millions of literatures focused on the study of functional relationships between genes. A network pathway contains a class of genes or gene products, which are represented as nodes, and the biological relationship between 2 nodes is represented as an edge (line). This kind of network is a graphical representation of the molecular relationships between genes or gene products. All the correlations or links are supported by at least one reference stored in the Ingenuity Pathways Knowledge Base, including literature, textbook, and canonical information. The intensity of the node color indicates the degree of regulation: the red represents up and the green represents down. The various shapes of nodes and labels of edges describe different functional gene product and the nature of the relationship between the nodes, respectively.

### 2.4. Immunoblots of Inflammatory Proteins

HepG2 cells were precultured in dishes for 24 h first and then starved by adding serum-free medium for additional 2.5 h. The cells were divided into 4 groups, which were a control group without treatment; a negative control group treated with 1 *μ*g/ml of LPS alone; two groups were treated with 20*μ*M of myricetin before exposure to LPS, or not. Every group were treated for 30 min. Cellular lysates were boiled for 5 min, equal amounts of which (40 *μ*g) were run on SDS-PAGE gel (10%) and transferred to PVDF membrane (Amersham Pharmacia Biotech). The membrane was incubated with specific primary antibody overnight at 4°C and incubated with respective secondary antibodies for 1 h. Immunoblot binds were detected by ECL system with the Image Quant LAS 4000 mini (GE Healthcare, Chicago, US). The relative amount of detected proteins was quantified by ImageQuant TL software.

### 2.5. Bio-Plex-Based Assays of Inflammatory Cytokine

HepG2 cells were precultured and starved accordingly to eliminate the effect of FBS. The cells were then treated with 10-20 *μ*M of myricetin for half hour before exposure to 1 *μ*g/mL LPS for additional 12 h. The 27 cytokines detection, including TNF-*α*, G-CSF, IFN-*γ*, IL-1*α*, IL-1*β*, IL-2, IL-4, IL-5, IL-6, IL-7, IL-8, IL-9, IL-10, IL-12(p70), IL-13, IL-15, IL-17, RANTES, Eotaxin, PDGF-BB, FGF basic, IP-10, MCP-1(MCAF), MIP-1*α*, MIP-1*β*, GM-CSF, and VEGF were performed by using Bio-Plex Pro Human Cytokine 27-Plex Panel kit (Bio-Rad Laboratories) and Bio-Plex cytokines assay system (Bio-Plex 200, Bio-Rad) according to the manufacturer's instructions and the results were analyzed by the Bio-Plex manager software (version 4.0).

### 2.6. Statistical Analysis

All the experimental data in the present study were repeated at least three or four times. Significances or differences of treated versus control were analyzed by the Student's t-test, and* p*<0.05 was considered significant.

## 3. Results

### 3.1. Gene Expression Profiling of Myricetin-Treated HepG2 Cells by GO Analysis

According to our previous study, we have performed basic analysis to the huge microarray data. Among the total 44K gene probes, 20*μ*M of myricetin upregulated the expressions of 143 genes (0.33% of total probes) and downregulated the expressions of 476 genes (1.08% of total probes) by greater than or equal to twofolds in HepG2 cells [18]. However, the comprehensive analysis of the data is scarce. Thus, we further utilize the free available tool, Gene Ontology, to analyze the basic characteristic of gene expressions in HepG2 cells by myricetin treatment. As shown in Table 1, three basic classes, including biological process, molecular function, and cellular component, were list out. In each class, we further list out the gene function subclasses with the ratio of significant regulated genes greater than 2% of the total, which includes 3, 5, and 16 subclasses, respectively.

Among biological process, signal transduction, metabolism, and lipid metabolism have been largely disturbed by myricetin treatment, with significantly regulated gene ratio of 7.27%, 4.68%, and 2.26%, respectively, which implies that myricetin has the potency on the regulation of signaling transduction and cellular metabolism, especially lipid metabolism. For instance, in signal transduction, expressions of* F2RL2, IGFBP1, ARHGAP26, PDE11A, ADM, SOS1, NF1, F2RL1, IL8, VDR, *and* TXNRD1* were identified to be linked to the biofunction of myricetin (Table S1). In cellular component, one of the notable affected subclasses is nucleus, containing the largest gene ratio of 19.22%. It involves several response element promoters of signaling pathways, such as ARE, NF-*κ*B, XRE, or AP-1, which are associated with oxidative stress response, inflammation, or xenobiotic metabolism. There are several gene expressions were remarkably altered in cellular component, including* BHLHB2, DIDO1, NDRG1, EID3, SORBS2, WDHD1, KIAA1429, SORBS2, JUN, *and* SLC2A4RG*, as shown in Table S2. Another important subclass is mitochondrion, with the differential gene ratio of 4.2%. Mitochondrion is critical in endothelial physiology and pathophysiology, and plays a prominent role on production of ATP, energy currency of the cell, through respiration, and then regulates the cellular metabolism [26, 27]. Myricetin was found to exert a crucial role on mitochondria function and keep the redox balance, which was evidenced by the differentially expressed genes after myricetin medication, such as* HSPA1A, GLS, PDK1, ABAT, SYNE2, ATP5S, *and* ETFDH* (Table S3). The significantly activated molecular functions were generally related to protein binding, DNA binding, kinase, and protein kinase activity, with the corresponding proportion of 27.3%, 10.02%, 3.39%, and 2.26%, respectively. Almost all the features of cells on their surfaces and interiors are based on diversity protein carrier in terms of tool-like receptor, facilitated glucose transporter, thioredoxin reductase 1, etc. Before that, many signal pathways associated to molecular function rely on DNA linking, the deliverance of genetic information, and the period is usually activated by protein kinases [28]. As shown in Table S3, a series of typical genes belonging to the above 4 subclasses disturbed by myricetin have been listed out, such as* SLC2A4RG, SLC22A3, SERPINE1, MAP3K13, ACVR1B, MAP3K8, ARHGAP26, BHLHB2, DMXL1, NCF2, HSPA1A/B, HMOX1, *FGF*R3, ATR, FGFR3, ACVR1B, WDHD1, HELLS, SOS1,* and* JUN. *These results indicated that myricetin has the potential to regulate molecular function, via regulation of gene expression pathways and modulation of signal transduction.

### 3.2. Gene Expression Profiling of Myricetin-Treated HepG2 Cells by IPA Analysis

Next, to further analyze the microarray data, we input the data into IPA system, and the gene expression profiling of myricetin-treated HepG2 cells has changed a little. As shown in Table 2, comparing the signals of myricetin-treated group with the control, the result revealed that the expressions of 15 genes were disturbed significantly by equal or greater than 4-fold, with upregulation of 8 genes and downregulation of 7 genes; the expressions of 36 genes were disturbed between 3-fold to 4-fold, with upregulation of 16 genes and downregulation of 20 genes; the expressions of 286 genes were disturbed between 2-fold to 3-fold, with upregulation of 56 genes and downregulation of 230 genes. Taken together, 337 gene expressions of the total 702 molecules (48%) exported by IPA were disturbed with fold changes over 2-fold.

### 3.3. Top Function and Canonical Pathway Analysis of Myricetin-Treated HepG2 Cells by IPA

To obtain a further investigation for the elucidation of the effects and underlying molecular mechanism of myricetin in HepG2 cells, the advanced analysis tool of IPA system was performed. In brief, IPA constructed the connection and relationship of the above significantly regulated genes, which also output the top 10 cellular functions and diseases altered by myricetin treatment in HepG2 cells with a* p*-value less than 0.05, as shown in Table 3. The most possible cellular function/disease of myricetin is the regulation of “cardiovascular disease, metabolic disease, and lipid metabolism,” which consists of 28 molecules with the score of 46. The secondary top cellular function/disease, “connective tssue development and function, skeletal and muscular system development and function, and tissue morphology” was significantly influenced by myricetin treatment as well, with 22 molecules and the score of 33. Besides, other cellular functions/diseases, such as “endocrine system disorders,” “cardiovascular system development and function,” “cellular assembly and organization,” “cellular movement,” “cancer,” “cell death,” and “hepatic system disease,” were also identified to be significantly disturbed by myricetin treatment. However, “cancer” was found to be the most frequently influenced disease with the occurrence of 4 times in top 10 functions, and “cell death” and “metabolism disease” were both mentioned 3 times there. The above result indicates that myricetin may exert its biofunction by the regulation of chronic disease related biomarkers and the representative signaling pathways. For instance,* MAP3K8*,* MAP3K13*,* NCF2*,* NF-κB (family)*,* NFATC2*,* NF-κB (complex),* and* NR2F2* were significantly disturbed by myricetin treatment, which are belonging to cancer related MAPK/NF-*κ*B inflammatory signaling pathway.

**Figure 1:**
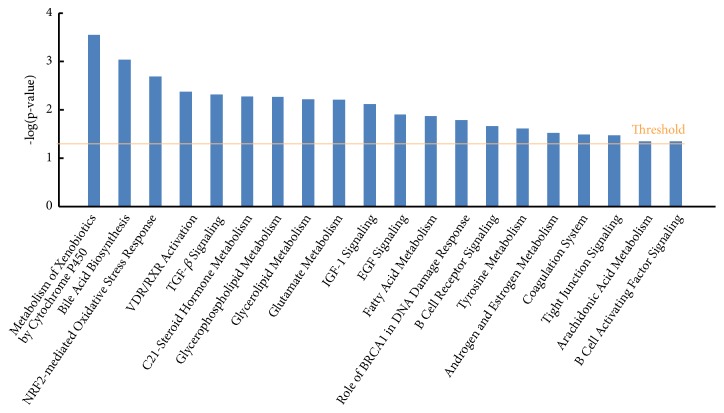
Top canonical pathway analysis of myricetin-treated HepG2 cells by IPA.

In addition to cellular function and disease, IPA system also listed out the significantly disturbed genes by distinct canonical pathways based on IPKB. As shown in Figure 1, IPA system outputs all the significantly affected signaling pathways by myricetin treatment with a* p-*value less than 0.05. Among the total 20 canonical pathways, Nrf-2-mediated oxidative stress response, TGF-*β* signaling, and B cell receptor signaling are strongly associated to chronic inflammation; metabolism of xenobiotics by cytochrome P450, bile acid biosynthesis, C21-steroid hormone metabolism, glycerophospholipid metabolism, glycerolipid metabolism, glutamate metabolism, IGF-1 signaling, and fatty acid metabolism are closely linked to metabolic disease, especially glucolipid metabolism dysfunction.

### 3.4. Network Analysis of Myricetin-Treated HepG2 Cells by IPA

Furthermore, IPA further built gene networks to connect key genes and enrich categories of diseases and functions, via the construction of the correlation between the significantly disturbed genes by myricetin treatment. We have listed out the top 1 network to further elucidate the cellular functions vividly and distinctly, corresponding to the top 1 cellular function and disease, as shown in Table 3.

**Figure 2:**
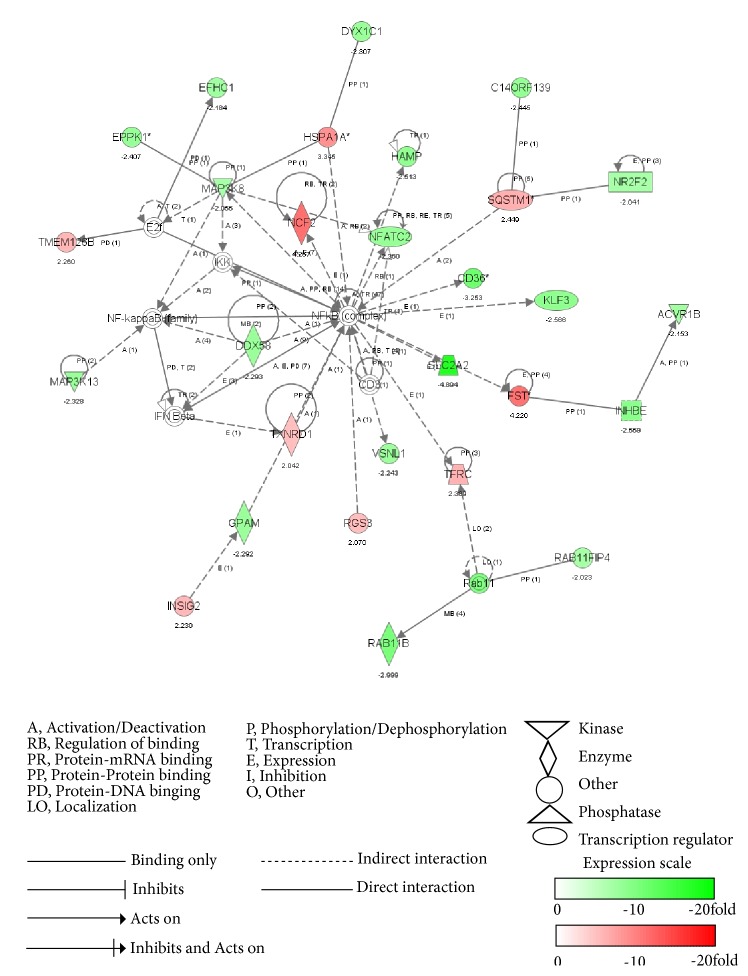
Top network analysis of myricetin-treated HepG2 cells by IPA. The dataset was analyzed by Ingenuity Pathway Analysis software. The node color indicates the expression level of the genes. Nodes and edges are displayed with various shapes and labels that present the functional class of genes and the nature of the relationship between the nodes, respectively.

As shown in Figure 2, top 1 network is related to cardiovascular disease, metabolic disease, and lipid metabolism. It consists of 35 genes, 28 of which are expressed differentially. It is worth noting that the cellular function of oxidative stress and inflammation response were vastly involved in this network. Among these genes,* SLC2A2 *(*solute carrier family 2 member 2*), also called* GLUT2*, is the most downregulated gene, with the reduction of 4.89-fold by myricetin treatment, and its expression is indirectly adjusted by* NF-κB *family. The reduction of* SLC2A2* expression suggested the inhibition of glucose absorption, which has a strong correlation to the glucose and lipid metabolic pathways [29]. Clinical data shows that* SLC2A2 (GLUT2)* mutations are the cause for Fanconi-Bickel syndrome, a rare autosomal recessive disease about carbohydrate metabolism dysfunction, and the tested patients showed typical characteristics such as glycogen storage disorders and proximal renal tubular nephropathy [30]. The most upregulated gene is* NCF2 *(*solute carrier family 2 member 2*), with the induction of 4.27-fold by myricetin treatment, and it is also indirectly adjusted by* NF-кB *family. NCF2 is reported to be associated with chronic disease, especially inflammatory chronic granulomatous disease [31]. Besides, multiple protein kinase genes related to inflammation are directly regulated by myricetin via modulation of inflammatory transcription factors. For instance,* MAP3K13 *and* MAP3K8*, the upstream protein kinases of NF-*κ*B pathway, displayed the decreasing expression folds of 2.06 and 2.33, respectively, where the expression of* MAP3K8 *was indirectly affected by* NF-κB*. Thus, we may speculate that inflammatory signal molecular and the upstream protein kinase would interact with each other.

What is more,* NF-κB *is the core of this network and monitors the activities of a series of inflammatory factors and the expressions of several typical antioxidant genes. Inflammatory genes* CD36* (3.25-fold),* KLF3 *(2.57-fold),* NFATC2 *(2.36-fold), and* ACVR1B *(2.15-fold) were downregulated indirectly by myricetin treatment via* NF-κB *pathway, while antioxidant genes* TXNRD1* (2.04-fold),* SQSTM1* (2.45-fold)*, TMEM126B* (2.26-fold), and* HSPA1A *(3.35-fold) were upregulated. All the above changes imply that myricetin could inhibit inflammation and activate the antioxidant chemoprevention to play a critical role in the cellular redox equilibrium. Two typical signaling pathways are found to be involved in the top 1 network, including B cell receptor signaling and Nrf2-mediated antioxidant pathway. B cell receptor signaling was reported to activate NF-*κ*B by PKC/TAK1 pathway [32].* NFATC2*,* MAP3K13,* and* MAP3K8* are belonging to B cell receptor signaling, and their downregulation reveals that myricetin can exert its anti-inflammatory effect via inhibition of B cell receptor signaling. Similarly, the upregulation of* SQSTM1* and* TXNRD1* in Nrf2-mediated antioxidant pathway indicates that myricetin can also inhibit inflammation via activation of Nrf2-mediated antioxidant pathway.

### 3.5. Real-Time qPCR Verification of the Specific Functional Genes

**Figure 3:**
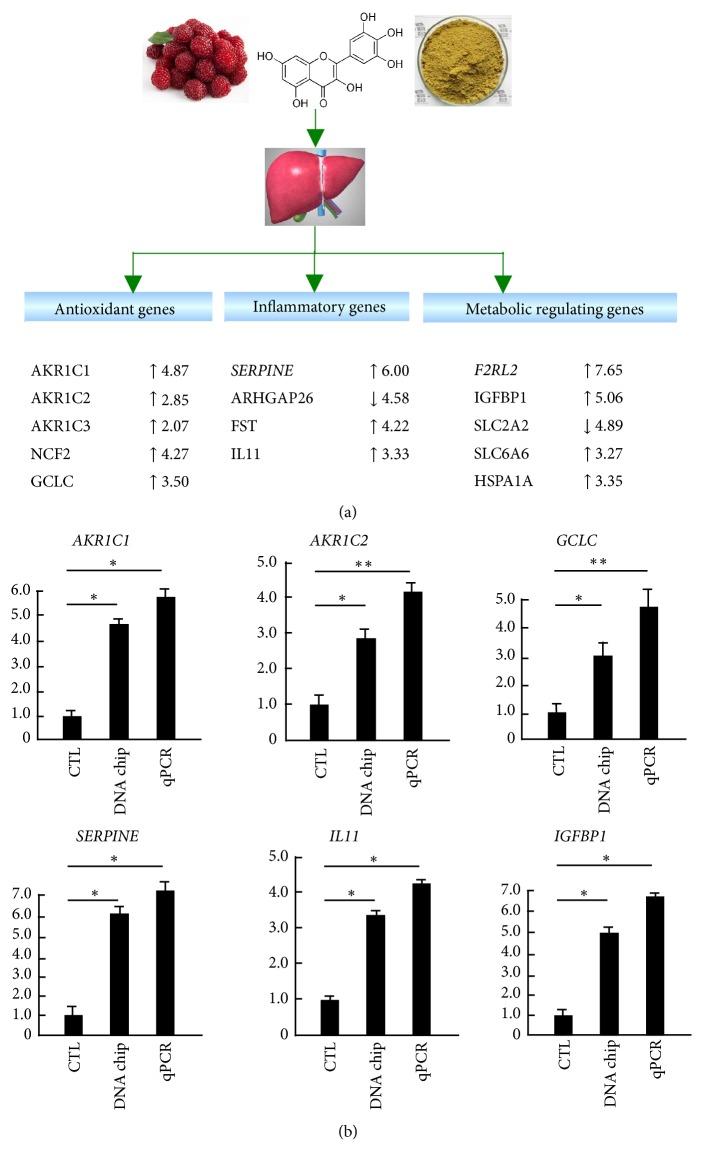
Real-time quantitative PCR verification of the specific functional genes identified by DNA microarray and IPA. (a) Three basic biofunction related genes filtrated by IPA. After DNA microarray, the whole data were input into ingenuity pathway analysis system. The genes classified to antioxidant genes, inflammatory genes, and metabolic regulating genes are displayed with more than twofold expression change by myricetin. (b) Real-time PCR verification data versus microarray data. HepG2 cells were pretreated with or without myricetin for 9 h. RNA extract and real-time PCR were described in Material and Methods. The result was expressed as the relative expression level. Each value represents the mean ± SD of three separate experiments, *∗*p < 0.05; *∗∗*p < 0.01 versus control, respectively. CTL, control; AKR1C, aldo-keto reductase family 1, member C; GCLC, glutamate cysteine ligase, catalytic subunit; SERPINE, serpin peptidase inhibitor clade; IL, interleukin; IGFBP1, insulin -like growth factor-binding protein 1.

To verify the microarray data and the above biofunction caused by myricetin in HepG2 cells, we performed RT-qPCR and compared the result of selective top 20 altered molecules in IPA to the raw gene chip data. As shown in Figure 3(a), we have selected typical significantly disturbed genes with their respective change fold from microarray data by myricetin treatment, including antioxidant genes* GCLC*,* AKR1C1/2/3* and* NCF2*, inflammatory genes* SERPINE, ARHGAP26, FST* and* IL11*, and metabolic genes* IGFBP1*,* F2RL2, SLC2A2/6A6,* and* HSPA1A*, which are all regulated by Nrf2 or NF-*κ*B signaling pathways. Then, we further verify the gene chip results of several selected key genes by RT-qPCR and make a comparison. As shown in Figure 3(b), RT-qPCR results have confirmed the accuracy of the gene chip results, and their changes are even bigger. These results indicate that myricetin can stimulate the expressions of antioxidant genes, inhibit that of inflammatory genes, and induce the expressions of metabolic regulating genes in HepG2 cells.

### 3.6. Bio-Plex Based Inflammatory Cytokines Assay by Myricetin Treatment in HepG2 Cells Induced by LPS

The above IPA analysis results reveal that inflammation is the main interface targeted by myricetin. However, these results just stay in mRNA level. To investigate whether myricetin indeed works on inflammation inhibition, and we conducted Western blotting and bio-plex inflammatory cytokine assay in HepG2 cells, which is a more suitable inflammation model. Cytokines are important in cell signaling and play a significant role in inflammation [33].

**Figure 4:**
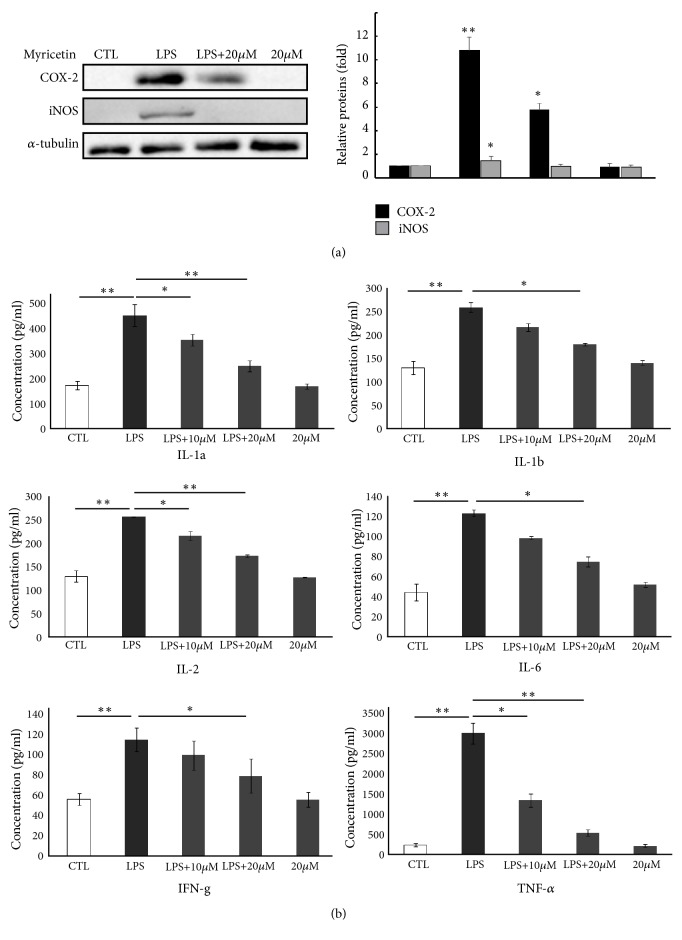
Influence of myricetin on the production of inflammatory proteins and cytokines. (a) Influence of myricetin on the production of iNOS and COX-2 protein. HepG2 cells (1×106 cells) were precultured for 24 h and starved in serum-free medium for 2.5 h. The cells were then treated with the indicated concentrations of myricetin for 30 min and then exposed to 1 *μ*g/mL LPS for 12 h. The proteins of iNOS, COX-2, and *α*-tubulin were detected by Western blotting with their antibodies, respectively. (b) Myricetin decreased the levels of multiple inflammatory cytokines in HepG2 cells. The levels of 27 kinds of cytokines were measured by multiplex technology and bio-plex assay and arranged in an order from high to low change in the experimental inflammatory HepG2 cells. The data represent mean ± SD of four mice. *∗*p < 0.05 and *∗∗*p < 0.01. CTL, control; IL, interleukin; IFN-*γ*, interferon-gamma; TNF-*α*, tumor necrosis factor.

Before starting, we firstly performed Western blotting of typical biomarkers of inflammation, iNOS and COX-2, to confirm the anti-inflammatory efficiency and the toxicity of myricetin. The results showed that iNOS and COX-2 expressions are both significantly decreased dose-dependently (Figure 4(a)) and 20 *μ*M of myricetin had no cytotoxicity on HepG2 cells, which is similar to our previous study[18]. Then, we applied 10-20 *μ*M of myricetin in bio-plex cytokines assay. As shown in Figure 4(b), 27 kinds of cytokines were detected by bio-plex system, just 9 of which were significantly inhibited by myricetin pretreatment, including TNF-*α*, IFN-*γ*, IL-1*α*, IL-1*β*, IL-2, and IL-6.

## 4. Discussion

In the present study, we conducted microarray and bioinformatic analysis to comprehensively describe the biofunction of myricetin in HepG2 cells, GO, and IPA analysis results provided some hints for the anti-inflammation, antioxidation, and intervention on metabolism of myricetin, which were partly verified by RT-qPCR. The function and canonical pathway analysis by IPA further confirmed that myricetin plays a crucial role on the regulation of redox signaling pathways and metabolic process, especially the inhibition on inflammation. Thus, we finally performed the proteomic analysis by the application of bio-plex cytokines assay, and the results indicated that myricetin does exert potent anti-inflammatory effect by secretion inhibitions of IL-1*α*, IL-1*β*, IL-2, IL-6, IFN-*γ*, and TNF-*α*.

Top molecules generated by IPA help us to focus on the most impossible biofunction rapidly. For example, top metabolic gene* SLC2A2* plays a key role in HIF-1-*α* signaling, which acts as the central system on regulation of metabolism. The concentration of HIF-1-*α* (and its subsequent activity) is regulated by NF-*κ*B-dependent regulation [34]. HIF-1 is overexpressed in many human cancers, and its overexpression promotes tumour growth and metastasis through its initial angiogenesis and regulation of cell metabolism to overcome hypoxia [35]. Thus, an inference can be made from the phenomenon that* SLC2A2* was involved in the HIF-1-*α* signaling, and its decreased expression by myricetin treatment implies that myricetin has the potency on the regulation of metabolic process, which is evidenced by the canonical pathway analysis of IPA. As shown in Figure 1, several metabolic pathways have been found to be associated with the metabolic regulation property of myricetin, including “metabolism of xenobiotics by cytochrome P450, bile acid biosynthesis, C21-steroid hormone metabolism, glycerophospholipid metabolism, glycerolipid metabolism, glutamate metabolism, IGF-1 signaling, and fatty acid metabolism.” These metabolic pathways were all significantly disturbed by myricetin treatment, indicating that myricetin possesses the ability of regulating glucolipid metabolism dysfunction.

In addition to the top 1 network associated with cellular function and disease, top 2 and top 3 networks are also found to be related to inflammation. As shown in Table 3, the top 2 and top 3 cellular functions and diseases are “connective tissue development and function, skeletal and muscular system development and function, and tissue morphology” (with a score of 33 and focus molecules of 22) and “endocrine system disorders, haematological disease, and metabolic disease” (with a score of 32 and focus molecules of 22). The cores of their networks are TGF-*β* and AP-1 (data not shown here), and their respective key molecules include* IL11*,* STAT*,* SORBS2*,* Sos*,* NEXN*,* SLC6A4*,* XRN1*, etc. Most of the inflammatory genes were significantly downregulated by myricetin treatment; however, a series of antioxidant genes, such as* SQSTM1*,* TXNRD1*,* HMOX1,* and* SLC family*, were found to be upregulated significantly, which reveals that myricetin plays a complex role on the balance between inflammation and antioxidation, the redox interface in HepG2 cells. Moreover, glucolipid metabolism was also found to be related to the redox status, for the disturbed expressions of* IGF2*,* IGF2BP2*,* IGFBP1,* and* IGFBP3*.

Our study is the first time to apply the inflammatory proteomic tool of bio-plex cytokines assay to investigate the inflammatory property of myricetin in HepG2 cells. In the total typical 27 cytokines, the secretions of IL-1*α*, IL-1*β*, IL-2, IL-6, IFN-*γ*, and TNF-*α* have been found to be inhibited by myricetin treatment. TNF-*α*, acting as a major member of tumour necrosis factor, is an important proinflammatory cytokine in the inflammatory response model. TNF-*α*, by means of binding to its receptor, can regulate NF-*κ*B expression by modulation of TRAF2 and RIP [36]. Therefore, the significant decrease of TNF-*α* expression indicates that the remission of inflammatory response is caused by myricetin treatment through the inhibition of NF-*κ*B activity, which is evidenced by other studies [37]. IL-6 and IL-1 are also important proinflammatory cytokines, and they were found to be inhibited remarkably by myricetin treatment in the present study. As reported, LPS can activate NF-*κ*B by stimulating monocyte MAPK signaling pathway and then promote the expression of IL-1 gene; besides, IL-1 can induce activation of p38 to promote the expression of NF-*κ*B in turn [38]. IL-6 is closely correlated with STAT3, and STAT3 plays a key role in cell proliferation and differentiation, so IL-6 can not only promote the development of inflammation but also accelerate the proliferation of normal cells and tumour cells [39]. Hence, NF-*κ*B signaling in the HepG2 cells was inhibited by myricetin because of the decrease of IL-6 and IL-1 productions. According to the result of proteomic assay of cytokines, spontaneously, the speculation that myricetin has a powerful effect on the inhibition of inflammation is obtained, which encourages* in vivo* studies to verify its possible health benefits.

## 5. Conclusions

Obviously, by the combined application of genomic microarray and proteomic cytokine assay, we not only comprehensively understand the multiple functions of myricetin, in the form of regulation on redox signaling pathways and metabolic process at mRNA level, but also verify its crucial and potent anti-inflammatory property at protein level. Therefore, myricetin exerts potent multiple biofunctions linked to redox balance and metabolic regulation, especially its remarkable anti-inflammatory activity. The present study could promote the application of nutritional intervention by myricetin in the research and development of functional food or special medical use food.

## Figures and Tables

**Table 1:** Gene expression profiling by GO analysis.

Class	Subclass	Ratio [%]
Biological process	Signal transduction	7.27
	Metabolism	4.68
	Lipid metabolism	2.26

Cellular component	Nucleus	19.22
	Extracellular	7.27
	Plasma membrane	6.79
	Cytosol	3.72
	Mitochondrion	4.20

Molecular function	Protein binding	27.30
	DNA binding	10.02
	Binding	7.59
	Kinase	3.39
	Protein kinase activity	2.26
	calcium ion binding	3.88
	nucleic acid binding	6.46
	transcription factor activity	4.36
	RNA binding	3.55
	nucleotide binding	9.69
	actin binding	3.55
	catalytic activity	5.33
	structural molecule activity	2.26
	protein kinase activity	2.26
	hydrolase	5.98
	Receptor activity	5.65

**Table 2:** Gene expression profiling of myricetin-treated HepG2 cells by IPA analysis.

Fold of Change		IPA data	
	Total	Numbers	Regulation
>4	15	8	up
		7	down

3<~<4	36	16	up
		20	down

2<~<3	286	56	up
		230	down

Total	337	80	up
		257	down

**Table 3:** Top 10 cellular functions and diseases altered by myricetin treatment in HepG2 cells.

	Molecules in Network	Score	Focus Molecules
Cardiovascular Disease, Metabolic Disease, Lipid Metabolism	ACVR1B, C14ORF139, CD3, CD36, DDX58, DYX1C1, E2f, EFHC1, EPPK1, FST, GPAM, HAMP, HSPA1A, IFN Beta, IKK, INHBE, INSIG2, KLF3, MAP3K8, MAP3K13, NCF2, NF-*κ*B (family), NFATC2, NF*κ*B (complex), NR2F2, Rab11, RAB11B, RAB11FIP4, RGS3, SLC2A2, SQSTM1, TFRC, TMEM126B, TXNRD1, VSNL1	46	28

Connective Tissue Development and Function, Skeletal and Muscular System Development and Function, Tissue Morphology	ADM, AGPAT9, Akt, BHLHB2, DCBLD2, HELLS, HSPG2 (includes EG:3339), Ige, IGF2, IGF2BP2, IL11, INPP5D, JAG1, Laminin, LCAT, Mek, NEXN, p70 S6k, PALLD, Pdgf, PDGF BB, PP2A, Ptk, RORA, Shc, SKAP2, SLC2A3, SLC6A4, SLC6A6, SORBS2, Sos, STAT, Tgf beta, TINAG, XRN1	33	22

Endocrine System Disorders, Hematological Disease, Metabolic Disease	Adaptor protein 2, AKR1B10, Ap1, AP2A1, BNIP3L, C5ORF34, CBR1, Ck2, Creb, DENND4A, hCG, HIP1, Histone h3, HMOX1, Igfbp, IGFBP1, IGFBP3, JUN, KLF5, LDL, MAFF, MAZ, Mmp, NRP1, P38 MAPK, PFKFB3, SMC4, SPP1, SULT2A1, SYNE2, tyrosine kinase, VDR, Vegf, VitaminD3-VDR-RXR, VRK1	32	22

Cardiovascular System Development and Function, Tissue Morphology, Amino Acid Metabolism	A1CF, AKR1C1, AKR1C2, AKR1C3, Angiotensin II receptor type 1, EGR1, ERK, ETS, F2RL1, FGFR3, Fibrin, FOS, G-Actin, GCLC, GCLM, GDF15, GNRH, JUN/JUNB/JUND, KLB, KLF4, LIMA1, NF1, NGF, NTN4, PLAUR, Rar, RASGRF2, Rxr, SCLT1, SERPINE1, SWI-SNF, T3-TR-RXR, Thyroid hormone receptor, TRA2A, Trans-1,2-dihydrobenzene-1,2-diol dehydrogenase	30	21

Cellular Assembly and Organization, Cellular Function and Maintenance, Nervous System Development and Function	14-3-3, ACTA1, ALB, ARHGAP26, ARHGDIA, ATP6V0A1, ATYPICAL PROTEIN KINASE C, CCAR1, CPLX1, F Actin, F2RL2, FGD4, GABBR1, Gsk3, Insulin, Jnk, MAP2K1/2, MARCKS (includes EG:4082), NEDD4L, Nfat, PARD3, PCYT1B, Pkc(s), Pld, PRLR, Rac, Raf, Ras, Ras homolog, RGNEF, SH3BP2, SOS1, TCR, TNS1, VAMP2	26	20

Cellular Movement, Dermatological Diseases and Conditions, Organismal Injury and Abnormalities	ADCY, AIM1 (includes EG:202), ALP, ARFGEF1, ARID1A, Calmodulin, Calpain, Cyclin A, Cyclin E, CYP1A1, ERK1/2, G alphai, Hdac, Histone h4, IL8, IL12, Interferon alpha, ITPR2, KNG1 (includes EG:3827), Mapk, MED13, MYLK, NDRG1, NRD1, PDZK1, Pka, Pkg, PRKAR1A, PTRF, Rb, RBL1, RNA polymerase II, SMARCA4, SUV420H1, THRB	24	18

Cancer, Cell Death, Reproductive System Disease	AASS, ANXA3, APPBP2, beta-estradiol, BICD1, BUB1, CCAR1, CHM, CORO2A, DIMT1L, EID3, FCGRT, geranylgeranyl pyrophosphate, GJB2, GRM3, HELLS, HRAS, IGFBP1, IREB2, KIAA2018, KLHDC2, MIRN29B2, MYC, NME5, NR3C1, PDK1, PTP4A2, RAB6A, RABGGTB, RASA4 (includes EG:10156), RPN2, SARDH, SELENBP1, SGK3, TXN	23	17

Cancer, Cell Cycle, Cell Death	ABAT, ADM, ALDH5A1, BMP15, CDKN2B, CEACAM5 (includes EG:1048), CENPE, CENPF, CMAS, CRK, FOXG1, FSH, FST, GCLC, GPRC5A, GRLF1, HNRPDL, INHA, KCNA5, KLF10, LACTB, LYVE1, MST1R, MTMR10, PCF11, PGRMC1, RB1, SEMA6B, SMAD5, SPAG4, TGFB1, THOC1, THOC2, thyroid hormone, TRIP11	21	16

Cell Death, Cancer, Cell Cycle	ACAP1, ADAM12, BBS7, C10ORF88, C16ORF57, C3ORF63, CALR, CD59, CLTC, CMIP, CUGBP1, DCP1A, F2RL2, KIAA1219, KIAA1429, KRT18, METTL7A, MME, PDIA2, PDIA3, PHACTR2, PPP1CA, PRLR, SH3BP4, SLC6A8, SMAD4, SRC, SSB, TFE3, TNS1, TOM1, TOM1L1, TUSC3, YWHAZ, ZFYVE16	21	16

Cancer, Hepatic System Disease, Liver Hyperplasia/Hyperproliferation	ATP5S, C3, CTBP1, CYP4B1, CYP4F11, EDA2R, ELK3, FGD4, FRY, GDF15, GPRC5A, LEFTY2, LRBA, MAP3K13, MAPK8, MAPK9, MINK1, MSLN, PEX1, PKP2, PRAM1, RAE1, RCOR3 (includes EG:55758), retinoic acid, RGL4, SH3BP5, SH3RF2, SMOX, SPAG9, SSBP3, STMN3, TAL1, TFF2, YWHAG, ZNF217	21	16

## Data Availability

The data used to support the findings of this study are available from the corresponding author upon request.

## Abbreviations

AP-1: Activator protein 1
GF: Growth factor
GO: Gene Ontology
I*κ*B: Inhibitory protein of NF-*κ*B
IRF3: Interferon regulatory factor 3
IL: Interleukin
IFN: Interferon
IPA: Ingenuity pathways analysis
LPS: Lipopolysaccharides
MAPK: Mitogen-activated protein kinase
NF-*κ*B: Nuclear factor-kappa B
Nrf2: Nuclear factor-erythroid 2 p45-related factor 2
NCF2: Neutrophil cytosolic factor 2 (65kDa, chronic granulomatous disease, autosomal 2)
RT-PCR: Real-time quantity polymerase chain reaction
SLC2A2: Solute carrier family 2 (facilitated glucose transporter), member 2
TGF-*β*: Transforming growth factor beta
TNF: Tumour necrosis factor.

## Supplementary Materials

Supplementary Materials Supplementary Table 1: the disturbed genes involved in signal transduction. Supplementary Table 2: the disturbed genes involved in nucleus and mitochondrion. Supplementary Table 3: the disturbed genes involved in DNA binding, protein binding, kinase, and protein kinase activity.
